# Supplementary material for: Challenges and facilitators in pathways to cancer diagnosis in Southern Africa: a qualitative study
Source: BMJ Open. 2025 Jun 25;15(6):e099296. doi: 10.1136/bmjopen-2025-099296 (PMC12198797; doi:10.1136/bmjopen-2025-099296)
Supplement: Supplementary file 2 [file bmjopen-2025-099296.reviewer_comments.pdf]

PEER REVIEW HISTORY

BMJ Open publishes all reviews undertaken for accepted manuscripts. Reviewers are asked to complete a checklist review form and are provided with free text boxes to elaborate on their assessment. These free text comments are reproduced below.

ARTICLE DETAILS

Title (Provisional)

Challenges and facilitators in pathways to cancer diagnosis in Southern Africa: A qualitative study

Authors

Day, Sarah; Arendse, Kirsten Deanne; Scott, Suzanne; Moyo, Melinda; Mzeche, Sunga; Guzha, Bothwell Takaingofa; Tegama, Natalie; Sills, Valerie Anne; Ras, Tasleem; Walter, Fiona M; Moodley, Jennifer

---

VERSION 1 - REVIEW

Reviewer 1

Name Watt, Melissa H

Affiliation University of Utah Intermountain Healthcare Department of Population Health Sciences, School of Medicine, University of Utah, 295 Chipeta Way, Salt Lake City, UT, 84108, USA., Population Health Sciences

Date 24-Feb-2025

COI None

This manuscript uses qualitative interviews with healthcare professionals in South Africa and Zimbabwe to examine the challenges and facilitators in pathways to cancer diagnosis. This is an important topic, as the delay to diagnosis often results in individuals having advanced disease at diagnosis and subsequently poorer health outcomes. The manuscript has some significant shortcomings, especially in how the results are synthesized and presented to allow for clear take-aways and actionable items for the global oncology community. I think these shortcomings can be overcome with a reorganization of the results section and some modifications to the discussion.

ABSTRACT

1. Missing a background section to state what is known and to justify the need for this research.

2. Results: See my comments in the results section

INTRODUCTION

1. The aim of the study (last paragraph) could be stated more clearly. Perhaps this needs to be broken into two sentences. I would also recommend adding a sentence about how these findings have the potential to impact research, clinical practice and policy.

METHODS

1. The comparison of the two country health systems as it relates to oncology is very wordy. Is there a way to put this in a table to make the takeaways more clear?

2. Sample: This lacks details. Were a certain number of healthcare facilities identified? What were the eligibility criteria of provider levels? Were any oncology specialists included in the sample?

3. The details about the interview guide should be pulled out as a separate paragraph. It does not make sense that details about the translation and transcription comes before the details about the interview guide.

4. For data analysis, please clarify if you used some type of deductive framework for your codebook. For example, did you organize codes by levels (individual, facility, etc) or by whether something emerged as a barrier or facilitator?

RESULTS

1. In the text and table 2, it is not clear what is meant by "cancers covered" when you're referring to primary health care. Were these PHC facilities providing oncological treatment? Or do you mean whether they have any diagnostic capabilities? This should be made more clear in the methods and results.

2. The results are not presented in a way that make it easy to take the findings away. The use of only two domains (Patient-level; Healthcare provider and system factors) under neglects the true multi-factoral influences that emerged. I would recommend that you look at the social-ecological framework as a way to organize the emerging themes.

3. In the individual level, the theme "refusal, absence and delays" is pointing to the problem, but not the drivers. In your text, you mention many of these drivers (e.g., fear, lack of trust in the health care system, mobility). I would recommend you revisit your data so that you can present these barriers.

4. The challenge of being able to contact trace patients (and the solution of CHWs) seems like it should be at the facility level

5. If you're presenting barriers, then "training needs" should be "training gaps"

6. The title states "barriers and facilitators" but this is not how the results are presented. If you want to frame everything as barriers, then I would reword the title.

DISCUSSION

1. After revising the results, the discussion should be updated accordingly

2. The second paragraph talks about the differences between Zimbabwe and South Africa. These differences were not discussed in the results section – I would suggest adding them there so that the contextual differences are clear.

3. The paragraph on CHWs speaks to the potential for CHWs to help patients navigate a complex and fragmented health system. But in fact, CHWs can also help patients to overcome other barriers such as trust in the health system, preference for traditional medicine

4. The paragraph on mHealth seems out of context. You should open that paragraph that with a clear connection to the data, and how you're thinking about mHealth in the context of cancer care to address some of the barriers that your study identified.

5. Only one limitation of the data is mentioned. I would recommend that the authorship team reflect on other limitations of this data and the conclusions that they draw.

6. The conclusion needs to be stronger. How does this study contribute to the literature and broader field? What are the "calls to action" for the research, clinical practice and policy communities in order to improve cancer care outcomes.

**Reviewer**	**2**
**Name**	**Mackintosh, Maureen**
**Affiliation**	**The Open University, Economics**
**Date**	**08-Apr-2025**
**COI**	**None**

This article addresses an important topic of rising concern among African clinicians, health workers and civil society groups: slow diagnosis of cancer, implying that many cancer patients are treated only at late stages, or indeed not at all. The article records welcome in-depth discussions with health professionals at primary and secondary/tertiary levels in South Africa (Eastern and Western Cape) and Zimbabwe (Harare and Bulawayo).

The methods are clearly described with one exception I wish to highlight here. It appears - but I have been able to find it clearly stated - that all the interviews are with public sector health workers. Yet the contextual presentation and the Discussion rightly emphasise that the health systems in both countries are complex, with both public and private provision and, in Zimbabwe, the leaching of HCWs from public to private is explicitly noted. There is a finding that patients move from private to public for lack of funding. In this context, the research question and methods should clarify (a) that the interviews are all in the public sector (b) why that decision was made. The implications could also be picked up in the Discussion.

In the Discussion, the authors refer to the usefulness of comparative analysis between the two countries (p.15). However, in the presentation of results, there is no explicit comparison of the responses in the two countries. The situation for HCWs in Zimbabwe is likely to be particularly difficult - as indeed the Discussion suggests - so the article would be strengthened by more indication within the results of differences between the two countries (despite the relatively small scale of the study).

Finally, a couple of more specific comments:

In the results, and Discussion, relating to training: from other work, and from one of the quotations here, it is clear that not only training but also recurrent practice is required to sustain initial diagnostic skills. However the discussion largely focuses on training and retraining: I would have liked to see a little more recognition of the associated need for regular supervised use of skills (e.g. on p.12).

Presentation of quotations: two quotations on p.10 use exactly the same phrasing but are attributed to different nurses. This can happen when responses are translated into standard English phraseology, but check that there is not a mistake here?

In the presentation of patient-level factors, the authors rightly emphasise that these are health professionals' perceptions only. However, interviews with cancer patients do evidence the extent to which patients' own delays may also be a reaction to poor response by the health system: in other words, the patients' and health system factors interact strongly. The article does mention issues such as trust and migration, but the discussion perhaps underplays the role of health system responses to patients in generating patients own delays and confusions? The two sets of factors are not as independent as separate presentation of them tends to imply?

Finally, please ensure that the Discussion recognises the limitation arising from the fact that that the experiences conveyed are of the public sector health workers only?

VERSION 1 - AUTHOR RESPONSE

Reviewer 1	
This manuscript uses qualitative interviews with healthcare professionals in South Africa and Zimbabwe to examine the challenges and facilitators in pathways to cancer diagnosis. This is an important topic, as the delay to diagnosis often results in individuals having advanced disease at diagnosis and subsequently poorer health outcomes. The manuscript has some significant shortcomings, especially in how the results are synthesized and presented to allow for clear take-aways and actionable items for the global oncology community. I think these	Thank you for your review and invaluable comments.

shortcomings can be overcome with a reorganization of the results section and some modifications to the discussion.	
ABSTRACT<br>1. Missing a background section to state what is known and to justify the need for this research.	We have followed the BMJ Open submission guidelines and included specified sections, as applicable, which does not include a background section. Information on what is known and the justification is included in the Introduction section.
2. Results: See my comments in the results section	Thank you, we have responded in the results section.
INTRODUCTION<br>1. The aim of the study (last paragraph) could be stated more clearly. Perhaps this needs to be broken into two sentences. I would also recommend adding a sentence about how these findings have the potential to impact research, clinical practice and policy	Thank you. We have now broken the aim of the study into two sentences (p. 5). It now reads:<br>"This study therefore aimed to explore primary, secondary and tertiary level HCWs experiences, barriers and facilitators in managing patients with symptoms of possible breast, cervical or colorectal cancer in two Southern African countries with varying health system challenges. The study took place in SA (in the medium human development index (HDI) category (0.709))28 and Zimbabwe (in the low HDI category (0.550))."
METHODS<br>1. The comparison of the two country health systems as it relates to oncology is very wordy. Is there a way to put this in a table to make the takeaways more clear?	Thank you. The two country's health systems pathways to diagnosis are not dissimilar. The primary difference is how user fees are setup. In Zimbabwe, patients are also required to use private laboratories for pathology. We have restructured the paragraphs to facilitate a clearer read.
2. Sample: This lacks details. Were a certain number of healthcare facilities identified? What were the eligibility criteria of provider levels? Were any oncology specialists included in the sample?	The eligibility of the sample is described in the Sample section. We have added further detail on how potential participants were identified and clarified that they were HCWs in PHC, secondary and tertiary public health facilities (p. 6).<br><br>"Potential participants were eligible for inclusion if they were staff with experience of working with patients who presented with symptoms of possible breast, cervical or colorectal cancer. HCWs were recruited from selected primary, secondary and tertiary level public health facilities. We used a matrix to purposively sample participants across the four regions, according to the level of their region, healthcare level, and job role, aiming for at least 12 primary care HCWs and at least 7 secondary/tertiary HCWs in each

	country. Potential participants were recruited from health facilities identified for the health facility assessment component for the AWACAN-ED[30] study. Potential participants were recruited from health facilities identified for the health facility assessment component for the AWACAN-ED[30] study. PHC facilities were sampled purposively, ensuring that a mix of rural and urban facilities were represented in the study sample. All secondary and tertiary facilities within the designated referral pathway were included."<br><br>We did not include oncologists as we examined pathways to diagnosis. Oncologists within these settings are involved post-diagnosis.
3. The details about the interview guide should be pulled out as a separate paragraph. It does not make sense that details about the translation and transcription comes before the details about the interview guide.	As suggested, the details about the interview guide have been pulled out as a separate paragraph and moved up to before details on transcription and translation (pp. 6-7).
4. For data analysis, please clarify if you used some type of deductive framework for your codebook. For example, did you organize codes by levels (individual, facility, etc) or by whether something emerged as a barrier or facilitator?	As suggested, we have clarified how the coding was done (p. 7): "Using the Model of Pathways to Treatment, data were coded deductively using the Diagnostic Interval factors. We used secondary tags to code for primary, secondary and tertiary levels of care. Sub-codes were inductively coded."
RESULTS<br>1. In the text and table 2, it is not clear what is meant by "cancers covered" when you're referring to primary health care. Were these PHC facilities providing oncological treatment? Or do you mean whether they have any diagnostic capabilities? This should be made more clear in the methods and results.	We have changed "cancers covered" to "cancers discussed" in the workshops to provide more clarity (p. 9). The PHC facilities do not provide oncological treatment, which is beyond the scope of this study, but assess patients with possible cancer symptoms for referral up to secondary or tertiary facilities with diagnostic capabilities.
2. The results are not presented in a way that make it easy to take the findings away. The use of only two domains (Patient-level; Healthcare provider and system factors) under neglects the true multi-factoral influences that emerged. I would recommend that you look at the social-ecological framework as a way to organize the emerging themes.	Thank you for this suggestion which we have considered. However, we do not think retrofitting the study to the socioecological model would strengthen the paper.<br><br>During analysis, we used the Model of Pathways to Treatment to frame our results, specifically focusing on patient and health system and provider factors that influence the Diagnostic Interval. In light of the reviewer's suggestion, we have focused on how other components of the health system,

	including broader contextual factors, finance and resources and trust, are embedded in patient- and health system and provider factors.
3. In the individual level, the theme “refusal, absence and delays” is pointing to the problem, but not the drivers. In your text, you mention many of these drivers (e.g., fear, lack of trust in the health care system, mobility). I would recommend you revisit your data so that you can present these barriers.	On page 4, in the Introduction, we have added a definition on each contributing factor in the Model of Pathways to Treatment:<br><br>“Patient-level contributing factors talk to both individual, social, cultural and contextual factors that impact each interval.[15] The health system and provider contributing factors represent healthcare service aspects impacting both patient and provider decision making and behaviour, and disease factors are the physiological aspects of the particular cancer.[15]”<br><br>Specifically, we have highlighted that patient-level contributing factors are not only factors that lie ‘within’ the individual, but how the individual is impacted by broader social, cultural and contextual realities.<br><br>Throughout the theme “refusal, absence and delays” we have added more detail on the broader inequitable contextual factors that are drivers of patient behaviour. We have added some more data where HCWs talk about other facilities who are reported to not be giving optimal care. We have written on page 12:<br><br>“Some participants did not offer in-depth reflections on how health provider attitudes could result in patients refusing procedures and missing appointments. Some participants shifted blame onto the patients, perceiving that patients do not take responsibility for their own health. Other participants spoke about how facilities, at the same or different levels of healthcare, have long waiting periods, patients receiving poor treatment at the facility or experience stigma going the facility near their home, which could result in patients seeking care outside their communities, not seeking timely care, refusing procedures or missing appointments.”

	We have also included additional quotes to support these findings (see p. 12).
4. The challenge of being able to contact trace patients (and the solution of CHWs) seems like it should be at the facility level	Thank you. However, not all CHWs are facility-based. Some CHWs are remunerated by government, other governments subsidise payment by NGOs, other are remunerated by NGOs. Therefore, CHWs are not strictly at a facility-level. We have left the results that talk to CHWs in the section 'Patients' refusal, absence and delays in attendance' as it talks to practical safety netting for patient-level factors.
5. If you're presenting barriers, then "training needs" should be "training gaps"	We have changed "needs" to "gaps".
6. The title states "barriers and facilitators" but this is not how the results are presented. If you want to frame everything as barriers, then I would reword the title.	We have presented facilitators as well as barriers throughout the results. Sometimes the distinction between barriers and facilitators is not always clear, as in some situations something may be either or both.<br><br>For example, (1) we talk to the use of CHWs to trace patients, which addresses patient-level barriers, but the tracing can take additional time; (2) We discuss how a breast cancer clinic has addressed referral challenges through setting up an electronic referral system that addresses some broader contextual and health system factors which result in delayed referral.
DISCUSSION<br>1. After revising the results, the discussion should be updated accordingly	Thank you, we have responded in the results section.<br><br>In the discussion section, we have written (p. 18):<br><br>"There is a bidirectional relationship between patient-, provider-, and health system delays. Patient-level delays talk to broader contextual and health system factors, such as socio-economic deprivation, geographic inequalities, mistrust in the health system, long waiting times, HCW attitudes and contextually insensitive referral processes. Similarly, HCWs are constrained by broader health system inequities, such as insufficient resources and equipment, limited training and supervision opportunities, unstandardised referral processes and poor communication within and between facilities."

2. The second paragraph talks about the differences between Zimbabwe and South Africa. These differences were not discussed in the results section – I would suggest adding them there so that the contextual differences are clear.	Throughout the results, we have read through to check for country comparisons, and we have strengthened where possible. Each paragraph now indicates whether the findings are from one or both countries. We have also included additional quotes where needed so both countries are better represented.
3. The paragraph on CHWs speaks to the potential for CHWs to help patients navigate a complex and fragmented health system. But in fact, CHWs can also help patients to overcome other barriers such as trust in the health system, preference for traditional medicine	Thank you. We have added that CHWs can assist building health system trust on page 19.<br><br>“Furthermore, CHWs are important health system agents to (re)build community trust in the healthcare system as they have strong relationships and are well regarded within the communities they serve.”<br><br>“When CHWs make formal referrals for patients to health facilities, it could improve patients’ institutional trust and reduce incidence of missed appointments and refusal of diagnostic procedures.”
4. The paragraph on mHealth seems out of context. You should open that paragraph that with a clear connection to the data, and how you’re thinking about mHealth in the context of cancer care to address some of the barriers that your study identified.	Under the section “Design” we have added that the findings from this study (and others related to AWACAN-ED) will be used to inform the development of public and PHC provider toolkits to improve pathways to diagnosis in South Africa and Zimbabwe. The paper by Arendse et al., specifically uses the second part of the interview to analyse participants’ experiences and perceptions of the barriers and facilitators to digital technology use to support early cancer diagnosis in their daily practice. This paper is referenced in the procedure and results.<br><br>We have added a sentence at the beginning of the paragraph on eHealth to specifically link it to pathways to diagnosis: “eHealth tools can be used to enhance patient pathways to diagnosis through addressing referral pathway gaps and challenges and strengthen pre-existing interventions.”
5. Only one limitation of the data is mentioned. I would recommend that the authorship team reflect on other limitations of this data and the conclusions that they draw.	We have added the following limitations (pp. 20-21):<br><br>“This study had a few limitations. Firstly, this study provides HCW perceptions of patient-level factors, which may not entirely reflect patient experiences, beliefs and circumstances. Secondly, this study did not

	interview private healthcare workers and does not offer insight into referral pathways from private HCWs perspectives between the private and public healthcare sectors. Thirdly, the interviews in Zimbabwe were mostly conducted in local languages, which could risk loss of depth or misinterpretation of meaning during the translation process. To mitigate this, we used multilingual interviewers as opposed to interpreters and multilingual interviewers checked the translated transcriptions for accuracy and completeness. Fourth, due to budget constraints, the study was conducted in two Southern African countries and particular provinces within them which limits the generalisability of the findings to the region more broadly. Fifth, despite purposively sampling for different provider types and years of experience, recruitment was influenced by the practical limitations of the number of providers employed within the facility, their willingness to participate, and their availability. Recruitment was monitored throughout the data collection process, and recruitment strategy adjusted to meet recruitment targets.”
6. The conclusion needs to be stronger. How does this study contribute to the literature and broader field? What are the “calls to action” for the research, clinical practice and policy communities in order to improve cancer care outcomes.	We strengthened the conclusion, and it now reads (p. 21):<br><br>“This study found a complex interaction between patient-, provider-, and health system factors influencing the Diagnostic Interval. Improving the diagnostic interval for breast, cervical, and colorectal cancer in Southern Africa necessitates targeted strategies that addresses patient- and health-system related factors with consideration given to socio-cultural and economic contexts. Documenting, strengthening, adapting and expanding already existing contextually relevant best practices, such as patient tracing using CHWs, mHealth tools and referral mechanisms, to address identified roadblocks along the Diagnostic Interval can shorten pathways to diagnosis.”
Reviewer 2	

This article addresses an important topic of rising concern among African clinicians, health workers and civil society groups: slow diagnosis of cancer, implying that many cancer patients are treated only at late stages, or indeed not at all. The article records welcome in-depth discussions with health professionals at primary and secondary/tertiary levels in South Africa (Eastern and Western Cape) and Zimbabwe (Harare and Bulawayo).	Thank you for your review and invaluable comments.
The methods are clearly described with one exception I wish to highlight here. It appears - but I have been able to find it clearly stated - that all the interviews are with public sector health workers. Yet the contextual presentation and the Discussion rightly emphasise that the health systems in both countries are complex, with both public and private provision and, in Zimbabwe, the leaching of HCWs from public to private is explicitly noted. There is a finding that patients move from private to public for lack of funding. In this context, the research question and methods should clarify (a) that the interviews are all in the public sector (b) why that decision was made. The implications could also be picked up in the Discussion.	Thank you. We have revised and made it clear that all the interviews are with public sector HCWs in the Abstract, Introduction, Design and Sample Sections. We made this decision as the overall aim for the larger AWACAN-ED[30] study is to develop a public sector PHC provider referral tool.<br><br>In the Discussion section (p.18), we have also clarified: "This study focused on the public health sector, as the overall aim of the AWACAN-ED study is to develop tools to improve timely referral from PHC to secondary and tertiary facilities. Understanding how the complex intersection of patient-, provider- and health system factors impacts the Diagnostic Interval is imperative for the development of a contextually valid and sensitive PHC referral tool for Southern Africa."
In the Discussion, the authors refer to the usefulness of comparative analysis between the two countries (p.15). However, in the presentation of results, there is no explicit comparison of the responses in the two countries. The situation for HCWs in Zimbabwe is likely to be particularly difficult - as indeed the Discussion suggests - so the article would be strengthened by more indication within the results of differences between the two countries (despite the relatively small scale of the study).	Throughout the results, we have read through to check for country comparisons, and we have strengthened where possible. Each paragraph now indicates whether the findings are from one or both countries. We have also included additional quotes where needed to support our findings and so that both countries are better represented.
In the results, and Discussion, relating to training: from other work, and from one of the quotations here, it is clear that not only training but also recurrent practice is required to sustain initial diagnostic skills. However, the discussion largely focuses on training and retraining: I would have liked to	Thank you. We have included regular supervision in the discussion section. Page 20 now reads:<br><br>"This study identified a need for continuous HCW training and professional development as well as regular supervision to improve and

see a little more recognition of the associated need for regular supervised use of skills (e.g. on p.12).	sustain cancer symptom assessment, particularly at PHC level."<br><br>"However, interventions to improve diagnostic knowledge and skills cannot be done as once offs as HCWs require regular training and supervision.[81] Furthermore, this study found that HCWs learn from each other. Interventions which strengthen supervision capacity within health facilities can strengthen ongoing supervision and mentorship structures."
Presentation of quotations: two quotations on p.10 use exactly the same phrasing but are attributed to different nurses. This can happen when responses are translated into standard English phraseology, but check that there is not a mistake here?	We have reviewed the quotations on page 10 and could not find the two quotations which the exact same phrasing. We have double-checked the quotes and there is not mistake.
In the presentation of patient-level factors, the authors rightly emphasise that these are health professionals' perceptions only. However, interviews with cancer patients do evidence the extent to which patients' own delays may also be a reaction to poor response by the health system: in other words, the patients' and health system factors interact strongly. The article does mention issues such as trust and migration, but the discussion perhaps underplays the role of health system responses to patients in generating patients own delays and confusions? The two sets of factors are not as independent as separate presentation of them tends to imply?	Thank you. Throughout the results, we have written more on broader health system and contextual factors that impact the patient-level factors. In the introduction, we have also specified that patient-level factors are not about what is 'within' or 'belonging to' the patient, but about factors that specifically impact how patients can access and interact with the health system. We have also reflected on how the participants did not provide any insight into how their own attitudes impact the patient-level, and shift onus onto the patient, other facilities, and so forth.<br><br>In the discussion section, we have written (p. 18):<br><br>"There is a bidirectional relationship between patient-, provider-, and health system delays. Patient-level delays talk to broader contextual and health system factors, such as socio-economic deprivation, geographic inequalities, mistrust in the health system, long waiting times, HCW attitudes and contextually insensitive referral processes. Similarly, HCWs are constrained by broader health system inequities, such as insufficient resources and equipment, limited training and supervision opportunities, unstandardised referral processes and poor communication within and between facilities."

Finally, please ensure that the Discussion recognises the limitation arising from the fact that that the experiences conveyed are of the public sector health workers only?	Thank you. We have added the following to the limitations section (P. 20-21):<br><br>"Secondly, this study did not interview private healthcare workers and does not offer insight into referral pathways from private HCWs perspectives between the private and public healthcare sectors. "

VERSION 2 - REVIEW

Reviewer 1

Name Watt, Melissa H

Affiliation University of Utah Intermountain Healthcare Department of Population Health Sciences, School of Medicine, University of Utah, 295 Chipeta Way, Salt Lake City, UT, 84108, USA., Population Health Sciences

Date 30-May-2025

COI

The revision has significantly improved this manuscript. I have some remaining suggestions for the authors as they finalize for publication.

Pg 4, line 32: change "talk to" to "represent"

Pg 8, lines 7 and 9: should be "patient and public involvement"

Pg 12: This sentence is not clear "Some participants did not offer in-depth reflections on how their own attitudes and practices could lead to patients refusing procedures and missing appointments." I think you can merge this sentence with the following sentence.

Pg 18, line 16: "bidirectional relationship" should be changed to "bidirectional relationships"

Pg 18: The added text in the first paragraph needs some work, as it doesn't flow very well. This sentence is particularly confusing. What does "talk to" mean here? "Patient-level delays talk to broader contextual and health system factors, such as socio-economic deprivation, geographic inequalities, mistrust in the health system, long waiting times, HCW attitudes and contextually insensitive referral processes."

Pg 19, line 38: The first time you use "eHealth," be more specific about what you mean.

VERSION 2 - AUTHOR RESPONSE

Reviewer 1	
The revision has significantly improved this manuscript. I have some remaining suggestions for the authors as they finalize for publication.	Thank you, Dr Melissa Watt, for your invaluable reviews and time put into this manuscript. Your time and contributions have assisted in strengthening the manuscript.
Pg 4, line 32: change “talk to” to “represent”	We have changed “talk to” to “represent”.
Pg 8, lines 7 and 9: should be “patient and public involvement”	We have changed to “patient and public involvement”.
Pg 12: This sentence is not clear “Some participants did not offer in-depth reflections on how their own attitudes and practices could lead to patients refusing procedures and missing appointments.” I think you can merge this sentence with the following sentence.	We have changed the sentence to: “Some participants did not offer in-depth reflections on their own attitudes and practices, and shifted blame onto patients, perceiving that patients do not take responsibility for their own health, or reflected on how attitudes and practices at other facilities, at the same or different levels of healthcare, result in patients seeking care outside their communities, not seeking timely care, refusing procedures or missing appointments.”
Pg 18, line 16: “bidirectional relationship” should be changed to “bidirectional relationships”	We have changed to, “There are bidirectional relationships...”
Pg 18: The added text in the first paragraph needs some work, as it doesn’t flow very well. This sentence is particularly confusing. What does “talk to” mean here? “Patient-level delays talk to broader contextual and health system factors, such as socio-economic deprivation, geographic inequalities, mistrust in the health system, long waiting times, HCW attitudes and contextually insensitive referral processes.”	We have restructured and edited the paragraph. We have broken the paragraph into two. The section now reads:<br><br>“Improving timely diagnosis of breast, cervical and colorectal cancer in Southern Africa requires strategies and interventions that are responsive to both patient-related and health system delays and challenges in the pathway from symptoms to diagnosis.[6, 12, 13] This study focused on the public health sector, as the overall aim of the AWACAN-ED study is to develop tools to improve timely referral from PHC to secondary and tertiary facilities. Understanding how the complex intersection of patient-, provider- and health system factors impacts the Diagnostic Interval is imperative for the development of a contextually valid and sensitive PHC referral tool for Southern Africa.<br>This study examined patient-, HCW-, and health system factors that impacted the Diagnostic Interval. There are bidirectional relationships between patient-, provider-, and health system delays. Patient-level

	delays represent broader contextual and health system factors, such as socio-economic deprivation, geographic inequalities, mistrust in the health system, long waiting times, HCW attitudes and contextually insensitive referral processes. In line with other studies, this study found that patient-level delays in the diagnostic interval were due to lack of access to transportation, rurality and distance to healthcare facilities, preference for alternative medicines and availability and affordability of medical care.[27, 48] Similarly, HCWs are constrained by broader health system inequities, such as insufficient resources and equipment, limited training and supervision opportunities, unstandardised referral processes and poor communication within and between facilities."
Pg 19, line 38: The first time you use "eHealth," be more specific about what you mean.	We have added a definition of eHealth after the first mention:<br>"eHealth is the use of information and technology systems to assist health and health-related fields.[71]"
